# Tuning the performance of CAR T cell immunotherapies

**DOI:** 10.1186/s12896-019-0576-9

**Published:** 2019-11-29

**Authors:** Noah H. Richardson, Jordan B. Luttrell, Jonathan S. Bryant, Damian Chamberlain, Saleem Khawaja, Indira Neeli, Marko Radic

**Affiliations:** Department of Microbiology, Immunology and Biochemistry, University of Tennessee Health Science Center, 858 Madison Avenue, Memphis, TN 38163 USA

**Keywords:** Immunotherapy, CAR T cells, Chimeric antigen receptors, Cancer, Autoimmunity

## Abstract

**Background:**

Simultaneous advances in gene editing, T cell engineering and biotechnology currently provide an opportunity for rapid progress in medicine. The approval of chimeric antigen receptor (CAR) T cell therapies by the US Food and Drug Administration (FDA) and the European Commission have generated substantial momentum for these first-in-class therapies to be used in patients with B cell malignancies.

**Main body:**

Considerable efforts focus on improved outcomes and reduced side effects of the newly approved therapies. Using innovative strategies, researchers aim to extend CAR T cell use to tackle difficulties inherent in solid tumors. Efforts are underway to broaden the applications of CAR T cells, and the strategy has been successful in chronic viral infections and preclinical models of autoimmunity. Research is in progress to generate “off-the-shelf” CAR T cells, an advance, which would greatly increase patient availability and reduce treatment cost.

**Conclusions:**

In this thematic review, we highlight advances that may help develop genetically engineered cells into a new category of medical therapies.

## Background

The remarkable success of CAR T cells in cancer patients, who had failed to respond to standard treatments, has captured the attention of researchers and the public at large [1]. The emergence of CAR T cells as therapeutic options with proven efficacy for B cell cancers is bolstered by the complete remission seen in most patients and the years of sustained efficacy that are possible. Potentially serious side effects, although deserving of continued attention, are largely transient and manageable with appropriate care and follow-up [2]. Below, we focus on how T cells can acquire genetic instructions to seek and destroy cancer cells. One particularly successful approach, which first showed efficacy in B cell leukemia, targets CD19, a B cell surface receptor expressed throughout most of B cell development [3, 4]. Viral vectors deliver the CAR transgene to a patient’s T cells. Upon integration into the host cell genome, the CAR gene encodes the chimeric receptor, which consists of a compact, extracellular targeting domain and additional transmembrane and cytoplasmic domains. The targeting domain usually derives from an antibody and mediates target cell binding, whereas the bi- or tri-partite cytoplasmic domain mediates T cell proliferation, differentiation, and, upon binding to a CD19-expressing B cell, promotes cell killing. Long-term engraftment of cytotoxic, cancer-suppressing T cells is achievable by the judicious design of cytoplasmic activation motifs. Accordingly, CAR T cells can eradicate even highly advanced B cell malignancies.

The currently approved CAR T cell therapies require autologous (patient-derived) T cells as recipients for the synthetic gene. However, the need for patient T cells as the starting point of the CAR therapy represents an obstacle to the broader application of the treatment. Only highly specialized research hospitals can apply the treatment, and patients with few or impaired T cells may be poor candidates for the treatment. In addition, variations in T cell phenotype, rates of viral transduction and cell culture conditions may differ from patient to patient and affect outcomes. Each of these hurdles have now begun to be surmounted. More advanced designs, which include multi-component CARs, inducible CAR expression and regulated CAR T cell activity, are in various stages of testing and implementation in cell culture, animal models of cancer, or trials in humans [5]. Here, we highlight recent advances that became possible by the creative application of genome editing to cell therapy.

### Methods for Germline editing

**Fig. 1:**
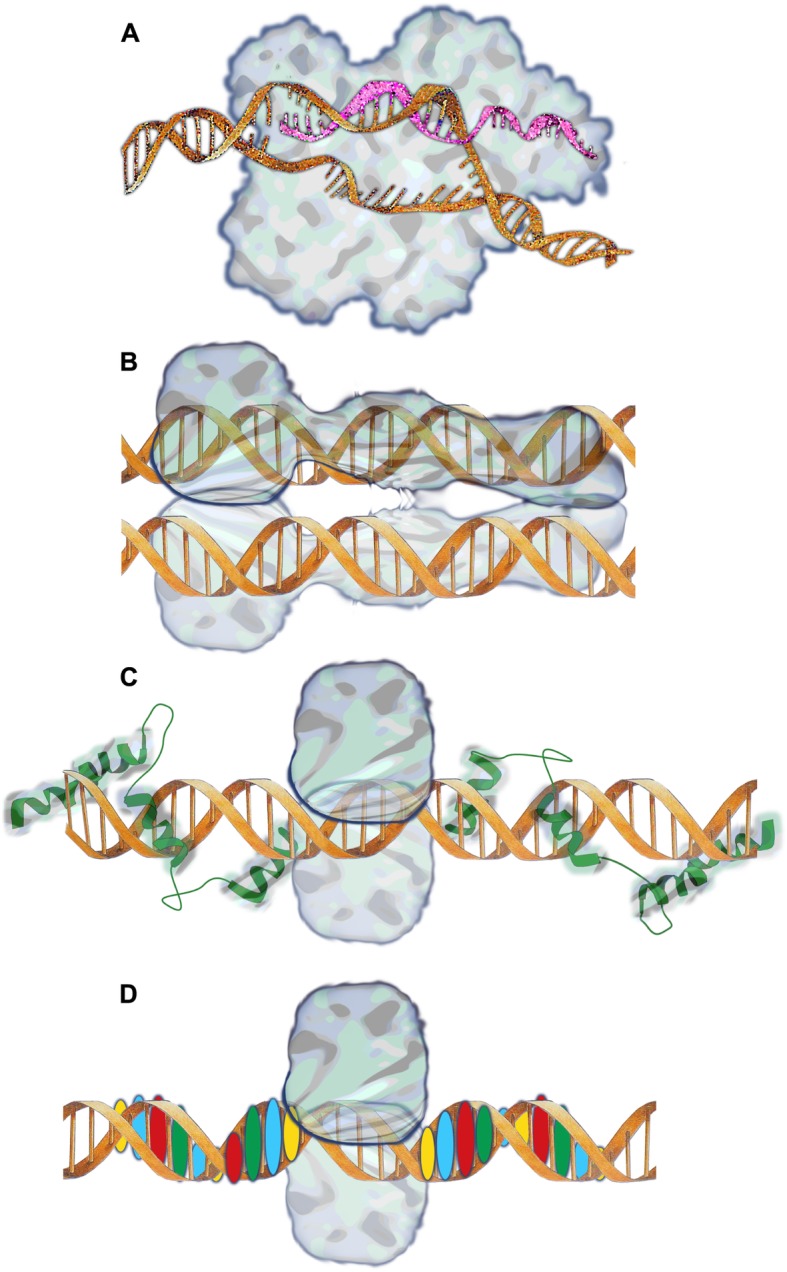
Diagrams of different approaches to genome editing. **a** CRISPR/Cas9 uses guide RNA (gRNA) to hybridize to a specific site in the genome and cleave the genomic DNA. **b** The Sleeping Beauty transposon aligns terminal repeat DNA sequences with target DNA prior to DNA cleavage and break repair to generate a T/A dinucleotide repeat at the site of initial cleavage. **c** Zinc finger (ZF) nuclease is shown with 3 finger domains recognizing either half site. **d** TALEN is shown to consist of nuclease (N) and protein domains that each recognize a unique base pair. The images are original depictions, not intended as precise molecular models of the proteins and nucleic acids involved in the reactions

Various approaches are available to modify the germline of somatic cells at sites of interest (Fig. 1). These range from the now pervasive use of CRISPR/Cas9, a bacterial nuclease that cleaves the DNA of infecting phage [6], to Sleeping Beauty, a modified version of a fish transposon [7], and to entirely engineered site-specific nucleases, such as zinc-finger arrays linked to restriction enzyme cleavage domains [8] and the transcription activator-like effector nuclease (TALEN) [9]. The cell activates its own DNA repair capacity to repair the double-stranded DNA breaks that the exogenous nuclease creates. The process relies on non-homologous DNA end joining and homology-directed repair, which often are error-prone. As result, the cleavage site is often repaired with insertions or deletions of one or more base pairs, which may interrupt an open reading frame. Investigators can produce larger insertions or deletions by generating DNA breaks at two genomic locations and bridging the sites by introduction of new or mutant DNA fragments. Transposon-based gene editing differs in that it relies on a transposase that can both cut and reseal the genome.

The gene editing methods differ in the complexity and speed of design and implementation: For site selection, design and production of new variants, CRISPR guide RNA (gRNA) offer a more efficient and versatile solution, whereas zinc fingers and TALENs are more intricate to work with. Each of these methods follows different criteria for the selection of suitable cleavage sites, yet entails the likelihood of additional events that potentially impact the host cell function by introducing unintended mutations. Of interest to the discussion below, the likelihood of genotoxicity is proportional to the concentration and length of time that a nuclease activity remains present in cells [10]. Thus, methods to bring mRNA or nuclease protein transiently into cells are available, such that they achieve the intended result, yet the enzymatic activity dissipates quickly upon editing of the host cell genome. Importantly, a single CRISPR experiment can accomplish simultaneous editing events at several sites in the genome [11].

### Tunable aspects of CAR therapy

**Fig. 2:**
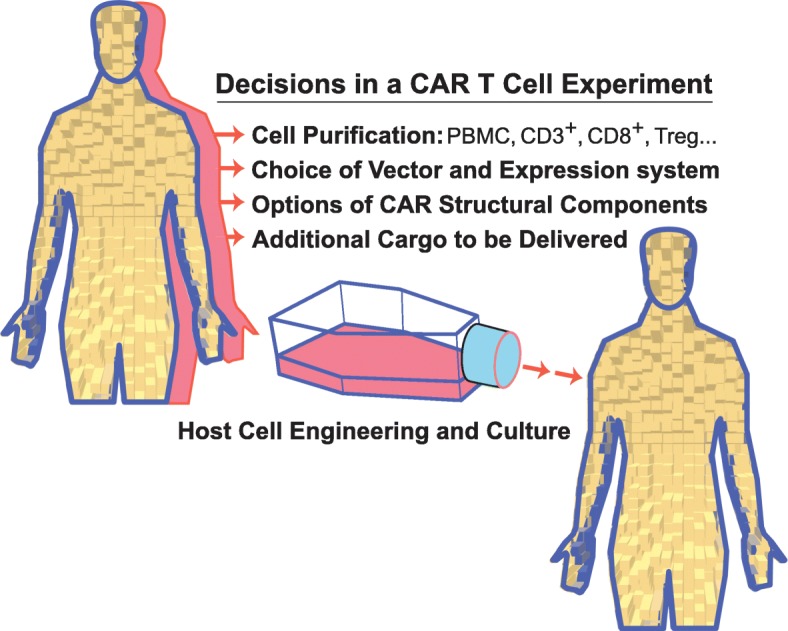
Aspects of CAR T cell culture and modifications that may be suited to different applications. A typical experiment that involves CAR T cells should consider various aspects of target cell populations, vector and expression system alternatives, structural aspects of the chimeric receptor protein and whether additional cargo should be delivered along with the CAR to the recipient cells. Importantly, gene editing of the CAR T cells may be a critical component of the design process. This is an original diagram

The decisions that arise in planning a typical CAR approach for cancer therapy are outlined in Fig. 2. The protocol involves the isolation of a sample of peripheral blood from the patient, which provides the starting point for the ex vivo portion of the procedure. An initial aspect of the procedure depends on the degree of cell purification that is intended before viral transduction. CAR transgene transduction may use total mononuclear cells, enriched CD3^+^ T cells, or purified cytotoxic CD8^+^ T cells. A trade-off between cell yield and efficacy drives this decision. Subsequent options include the choice of CAR specificity, overall structure of the CAR fusion protein, and expression system. Currently, most studies use a second or third generation CAR (using two or three cytoplasmic signaling domains), which, in most cases, is delivered by lentivirus, although retrovirus delivery also offers certain benefits. In addition to the CAR structural gene, researchers have used the viral delivery vector to encode a variety of additional gene products. For example, vectors may express factors that enhance survival of CAR T cells in the patient, such as interleukins [12], or to include a “safety switch”, which could disable the CAR T cells, if the patient suffers unacceptably serious side effects. One method to disable CAR T cells is the use of an inducible caspase gene [13, 14] that can be activated should the therapy prove to be dangerous for the patient. Such safety measures are frequently included with more recent CAR T cell clinical trials.

A productive area of CAR T cell engineering involves the modification of the CAR T cells in ways that could be beneficial upon transplant into the patient. There are several important objectives that have been approached in this category. By gene editing (using methods outlined in Fig. 1), CAR T cells have been rendered safer, more resilient, and more active within the tumor microenvironment. For example, researchers have sought to block the major antigen presentation functions of CAR T cells, such that the cells will not be attacked by the host immune system [11]. Additional efforts have aimed at making CAR T cells capable of remaining active in the milieu of a solid tumor [15]. An important improvement of CAR T cell persistence in vivo was achieved by the introduction of a tethered IL15 cytokine along with its receptor onto the surface of CAR T cells, which could provide CAR T cells with memory phenotype and increased persistence within the tumor microenvironment [16]. As several cellular receptors serve to limit the excessive activation of an immune response, checkpoints exist to limit clonal proliferation. Tumors adapt these checkpoint signals and blunt the effects of tumor infiltrating lymphocytes. Examples of such receptors on T cells are PD-1 [17] and CTLA-4 [11], which act as checkpoint mediators.

Interestingly, important phenotypic changes may be brought about by T cell culture conditions. It was recognized that transduced T cell cultures can be expanded over 100-fold above the initial cell numbers. However, it was also reported that culture for over 10–14 days yields expanded T cells that are less well suited for immunotherapy [18]. Therefore, it was proposed that ex vivo culture should be limited to between 3 and 5 days before the CAR T cells are administered to a patient [18]. Notably, agonists or antagonists of signal transduction pathways may be useful additives to the culture medium because the treated T cells achieve a central memory phenotype, which increases the likelihood that T cells will resist exhaustion in vivo and continue to generate effector T cells over an extended time [19–21].

### New CAR designs and implementations

Starting in 2012, several laboratories observed that, if the endogenous T cell receptor (TCR) in CAR T cells is inactivated, the safety profile of the genetically modified T cells improves [7, 9, 22]. Thereby, the foundations were laid to develop lines of CAR T cells that are potentially suitable for use in allogeneic transplants. In additional studies, researchers discovered that the efficacy and performance of CAR T cells may be improved by insertion of the CAR transgene into the endogenous TCR locus [23]. Researchers conducted experiments in which either the TCR alpha (TRAC) or the TCR beta (TRBC) constant domain loci were selected for CAR transgene integration sites and both resulted in similar performance improvements.

The initial experiments used a variety of nucleases to induce gene editing, but over time, the ease of use and versatility of the CRISPR/Cas9 system prevailed in most laboratories [6]. Once robust methods for cell transfection with mRNA for the Cas9 enzyme (or other nucleases) were established, other endogenous loci were also inactivated, including the class I HLA, or the beta-2 microglobulin gene [6, 11]. In continuation, researchers turned their attention to the editing of genes that make CAR T cells susceptible to negative regulation in the tumor microenvironment. For these experiments, researchers used site-specific nucleases to inactivate PD-1, CTLA-4 and Fas [24], each of which may inhibit effector functions of T cells and thereby contribute to T cell suppression by tumor cells, which often express ligands that induce checkpoint regulation.

In general, the benefits of endogenous TCR inactivation include the lack of interference with CAR signaling and the reduced likelihood that the CAR T cells could lead to graft-versus-host responses. Conversely, deletion of checkpoint inhibitors promises to overcome one of the hurdles in the application of CAR T cells in solid tumors, an important objective in oncologic CAR therapies. One recent advance toward generating universal donor CAR T cells with increased resistance to tumor-induced immune suppression was achieved by researchers at the University of Pennsylvania. In their study, deletion of multiple T cell genes in the same cells was accomplished by the introduction of gRNAs as part of the lentiviral genome [11]. The U6 promoter drove expression of gRNAs, whereas the anti-CD19 CAR was expressed downstream from the EF1 alpha promoter/enhancer cassette. The Cas9 nuclease was added as mRNA or as protein to the cells by electroporation. These researchers successfully inactivated the endogenous TCR, HLA class I, Fas, PD-1 and CTLA-4 genes.

The introduction of gRNA together with mRNA for Cas9, when followed by flow sorting of the resulting population of cells, can help to produce nearly uniformly gene-edited progeny. Clearly, the CAR T cells that are recovered might still represent a germline mosaic in terms of complete gene disruption, CAR expression and second-site mutations [25]. Inclusion of guide RNA within the regulatory sequences of the CAR lentivirus improves targeting of the editing process to CAR T cells [26]. The goal of a “universal” CAR T cell source, however, will need to be combined with a highly accurate and comprehensive assessment of genome integrity [27].

### Beckoning CAR roadmaps

The last few years have seen a full blossoming of creative applications of the basic CAR T cell approach [28]. The field has seen the introduction of CAR transgenes into different cell lineages, such as natural killer cells (NK [29–31];) and regulatory T cells (Tregs [32];), the further modification of the engineered gene products [5], the testing of “off the shelf” CAR T cells [33], and the initial applications to fields outside of immuno-oncology (see below). Here, only examples that illustrate the breadth of the scientific advances are provided, rather than a complete accounting of the field.

Due to the success of the anti-CD19 CAR approach, further modifications of the CAR fusion protein have taken center stage. The expansion of CAR T cell specificity includes other B cell markers such as CD20 [34], CD22 [35], and B cell maturation antigen (BCMA [36];), but also surface markers that are predominantly expressed on specific cancer cells. Some of these have been combined, for example CD19 and CD20, in part to avoid the outgrowth of escape variants [37]. Additional CAR targets include HER2 [38], IL13Rα2 (glioblastoma [39];), MUC1 (variety of cancers, [40]), and B7-H3 (wide range of tumor types [41];). The basic structure of a second or third generation CAR has been altered in major ways, such that a TCR-associating chimeric protein was designed and tested [42]. An important advance was achieved by showing that two extracellular targeting domains can be connected to two separate signaling domains, such that cytotoxic activity is only induced if both targets are present on the same cell [43]. This modification, which separates signaling through CD3zeta from CD28 co-stimulation, allows the increased on-tumor specificity in situations where a unique tumor target is unavailable.

Modifications of CAR structure also involved minor, yet highly significant variations, such as the point-mutations of tyrosine residues in the CD3zeta tail of an anti-CD19 CAR [44]. The reduction in CAR signaling dramatically increased efficacy and persistence of CAR function, a result that confirmed previous studies from the Rosenberg lab [45, 46]. The important conclusion from these studies is that the signaling strength of the CAR cytoplasmic domain determines the capacity of the CAR T cells to maintain long-term persistence in the recipient. If signal transduction is too powerful, the CAR T cells will show potent activation but also a greater tendency toward exhaustion, a state in which activated T cells no longer respond to antigen.

Studies suggest that one critical determinant of CAR T cell efficacy is the capacity of CAR T cells to acquire a T central memory phenotype (T_CM_) in vivo. This characteristic enhances persistence in the host, supports extended capacity for cell division and favors differentiation into effector T cells [47]. One marker for T_CM_ is the abundant expression of CD62L, also known as L-selectin, which represents a useful indicator of the in vivo potential of CAR T cells. The potential of CAR T cells for T_CM_ function was assessed following growth under different culture conditions. Culture in the presence of Akt-1 inhibitor [19] or with RORgamma agonists [21] imbued CAR T cells with a durable memory phenotype and ensured their long-term in vivo engraftment and efficacy.

An important development in the field of CAR T cell therapy has been the extension of the basic immunotherapy principles to clinical situations in which a “reboot” of the immune system is desirable and potentially curative. Two major areas of immunology have seen efforts to employ CAR T cells to combat chronic infections and autoimmunity. For treatment of HIV infections, neutralizing antibodies to HIV were used as source of the extracellular binding domain for a newly designed CAR that showed promise in CAR T cell culture systems [48], although improvements in other aspects of the fusion protein and expression system still are deemed necessary [49]. Chronic hepatitis B infections may also become treatable with CAR T cells, as suggested by pre-clinical studies [50].

In pre-clinical studies of two autoimmune diseases, pemphigus vulgaris and systemic lupus erythematosus, CAR T cells showed remarkable efficacy in alleviating the manifestations of autoimmunity. To treat pemphigus vulgaris, desmoglein-specific B cells, the main culprits in this skin disorder, were targeted by CAR T cells offering portions of desmoglein as “bait” to bind and kill the B cells [51]. The success of this study encourages clinical trials in this, previously refractory, autoimmune disease. In a study from our lab, we applied anti-CD19 CAR T cells to a classic autoimmune disorder called systemic lupus erythematosus (SLE). In two strains of lupus mice, disease manifestations were stopped or reversed by treatment with standard anti-CD19 CAR T cells, reflecting the sustained persistence of CAR T cell function [52]. The efficacy of the CAR T cells mirrored the persistence of CAR T cells for over 1 year after infusion into the recipient animals. Consequently, the treated mice attained a near-normal life span. Studies such as these open the door to other applications in autoimmune disorders, in which B cells have defined contributions to pathogenesis.

### Commercial interest in CAR technology

With the approval of anti-CD19 CAR T cells for human therapy of B cell malignancies and with the impressive valuation of the initial companies that entered the CAR T cell biotechnology field, commercial interest in CAR technology companies rapidly increased. The first companies, Novartis and Kite, reported results from clinical trials that led to the FDA approval of CAR T cell products named Kymriah and Yescarta. Currently, there are over 200 CAR T cell clinical trials worldwide and over 40 biotechnology companies participating in some manner in the further development and testing of CAR T cell therapies. These include established pharmaceutical companies that have acquired smaller start-ups with the technical expertise in the field, but also growing and independent companies such as Atara Biotherapeutics, Bellicum Pharmaceuticals, Bluebird Bio, Cellectis, Fate Therapeutics, Lyell Immunopharma, Precision Biosciences, Sorrento Therapeutics and Ziopharm Oncology, among others. With the broad and growing interest of investors, and fueled by exciting discoveries in applying the new CAR T cell therapies in healthcare, expansion in this area of biotechnology is more than likely to continue.

## Conclusions

The exciting confluence of molecular biology techniques, engineering of immune cell types, and the successful targeting of cancer surface antigens opened the doors to the wider application of immunotherapies for a growing number of diseases that heretofore were untreatable. We are fortunate to experience the seamless and rapid transition between genetic analysis, molecular design, testing in animal models and application to the clinic. Within a few short years, creative and almost intuitive designs of new therapies are rapidly generating new data and justifying applications to a growing number of patients. Future challenges will be the development of safe and widely applicable immunotherapies that will bring prospects for the lasting cure of chronic, progressive and lethal disorders that have plagued mankind.

## Data Availability

All data were previously published and thus are available.

## Abbreviations

CAR: Chimeric antigen receptor
CD: Cluster of Designation
FDA: Food and Drug Administration
gRNA: Guide RNA
PD-1: Programmed Death 1
TALEN: Transcription activator-like effector nuclease
TCR: T- cell receptor

## References

[1] June CH, O'Connor RS, Kawalekar OU, Ghassemi S, Milone MC (2018). CAR T cell immunotherapy for human cancer. Science.

[2] Brudno JN, Kochenderfer JN (2019). Recent advances in CAR T-cell toxicity: mechanisms, manifestations and management. Blood Rev.

[3] Porter DL, Levine BL, Kalos M, Bagg A, June CH (2011). Chimeric antigen receptor-modified T cells in chronic lymphoid leukemia. N Engl J Med.

[4] Kalos M, Levine BL, Porter DL, Katz S, Grupp SA, Bagg A, June CH (2011). T cells with chimeric antigen receptors have potent antitumor effects and can establish memory in patients with advanced leukemia. Sci Transl Med.

[5] Chakravarti D, Wong WW (2015). Synthetic biology in cell-based cancer immunotherapy. Trends Biotechnol.

[6] Liu X, Zhao Y (2018). CRISPR/Cas9 genome editing: fueling the revolution in cancer immunotherapy. Curr Res Transl Med.

[7] Torikai H, Reik A, Liu PQ, Zhou Y, Zhang L, Maiti S, Huls H, Miller JC, Kebriaei P, Rabinovich B (2012). A foundation for universal T-cell based immunotherapy: T cells engineered to express a CD19-specific chimeric-antigen-receptor and eliminate expression of endogenous TCR. Blood.

[8] Torikai H, Reik A, Soldner F, Warren EH, Yuen C, Zhou Y, Crossland DL, Huls H, Littman N, Zhang Z (2013). Toward eliminating HLA class I expression to generate universal cells from allogeneic donors. Blood.

[9] Poirot L, Philip B, Schiffer-Mannioui C, Le Clerre D, Chion-Sotinel I, Derniame S, Potrel P, Bas C, Lemaire L, Galetto R (2015). Multiplex genome-edited T-cell manufacturing platform for “off-the-shelf” adoptive T-cell immunotherapies. Cancer Res.

[10] Gangopadhyay SA, Cox KJ, Manna D, Lim D, Maji B, Zhou Q, Choudhary A (2019). Precision control of CRISPR-Cas9 using small molecules and light. Biochemistry.

[11] Ren J, Zhang X, Liu X, Fang C, Jiang S, June CH, Zhao Y (2017). A versatile system for rapid multiplex genome-edited CAR T cell generation. Oncotarget.

[12] Krenciute G, Prinzing BL, Yi Z, Wu MF, Liu H, Dotti G, Balyasnikova IV, Gottschalk S (2017). Transgenic expression of IL15 improves Antiglioma activity of IL13Ralpha2-CAR T cells but results in antigen loss variants. Cancer Immunol Res.

[13] Budde LE, Berger C, Lin Y, Wang J, Lin X, Frayo SE, Brouns SA, Spencer DM, Till BG, Jensen MC (2013). Combining a CD20 chimeric antigen receptor and an inducible caspase 9 suicide switch to improve the efficacy and safety of T cell adoptive immunotherapy for lymphoma. PLoS One.

[14] Hoyos V, Savoldo B, Quintarelli C, Mahendravada A, Zhang M, Vera J, Heslop HE, Rooney CM, Brenner MK, Dotti G (2010). Engineering CD19-specific T lymphocytes with interleukin-15 and a suicide gene to enhance their anti-lymphoma/leukemia effects and safety. Leukemia.

[15] Akhavan D, Alizadeh D, Wang D, Weist MR, Shepphird JK, Brown CE (2019). CAR T cells for brain tumors: lessons learned and road ahead. Immunol Rev.

[16] Hurton LV, Singh H, Najjar AM, Switzer KC, Mi T, Maiti S, Olivares S, Rabinovich B, Huls H, Forget MA (2016). Tethered IL-15 augments antitumor activity and promotes a stem-cell memory subset in tumor-specific T cells. Proc Natl Acad Sci U S A.

[17] Rupp LJ, Schumann K, Roybal KT, Gate RE, Ye CJ, Lim WA, Marson A (2017). CRISPR/Cas9-mediated PD-1 disruption enhances anti-tumor efficacy of human chimeric antigen receptor T cells. Sci Rep.

[18] Ghassemi S, Nunez-Cruz S, O'Connor RS, Fraietta JA, Patel PR, Scholler J, Barrett DM, Lundh SM, Davis MM, Bedoya F (2018). Reducing ex vivo culture improves the Antileukemic activity of chimeric antigen receptor (CAR) T cells. Cancer Immunol Res.

[19] Klebanoff CA, Crompton JG, Leonardi AJ, Yamamoto TN, Chandran SS, Eil RL, Sukumar M, Vodnala SK, Hu J, Ji Y, et al. Inhibition of AKT signaling uncouples T cell differentiation from expansion for receptor-engineered adoptive immunotherapy. JCI Insight. 2017;2(23):e95103. 10.1172/jci.insight.95103 PMC5752304 29212954

[20] Urak R, Walter M, Lim L, Wong CW, Budde LE, Thomas S, Forman SJ, Wang X (2017). Ex vivo Akt inhibition promotes the generation of potent CD19CAR T cells for adoptive immunotherapy. J Immunother Cancer.

[21] Hu X, Majchrzak K, Liu X, Wyatt MM, Spooner CJ, Moisan J, Zou W, Carter LL, Paulos CM (2018). In vitro priming of adoptively transferred T cells with a RORgamma agonist confers durable memory and Stemness in vivo. Cancer Res.

[22] MacLeod DT, Antony J, Martin AJ, Moser RJ, Hekele A, Wetzel KJ, Brown AE, Triggiano MA, Hux JA, Pham CD (2017). Integration of a CD19 CAR into the TCR alpha chain locus streamlines production of allogeneic gene-edited CAR T cells. Mol Ther.

[23] Eyquem J, Mansilla-Soto J, Giavridis T, van der Stegen SJ, Hamieh M, Cunanan KM, Odak A, Gonen M, Sadelain M (2017). Targeting a CAR to the TRAC locus with CRISPR/Cas9 enhances tumour rejection. Nature.

[24] Ren J, Liu X, Fang C, Jiang S, June CH, Zhao Y (2017). Multiplex genome editing to generate universal CAR T cells resistant to PD1 inhibition. Clin Cancer Res.

[25] Knipping F, Osborn MJ, Petri K, Tolar J, Glimm H, von Kalle C, Schmidt M, Gabriel R (2017). Genome-wide specificity of highly efficient TALENs and CRISPR/Cas9 for T cell receptor modification. Mol Ther Methods Clin Dev.

[26] Georgiadis C, Preece R, Nickolay L, Etuk A, Petrova A, Ladon D, Danyi A, Humphryes-Kirilov N, Ajetunmobi A, Kim D (2018). Long terminal repeat CRISPR-CAR-coupled “universal” T cells mediate potent anti-leukemic effects. Mol Ther.

[27] Giannoukos G, Ciulla DM, Marco E, Abdulkerim HS, Barrera LA, Bothmer A, Dhanapal V, Gloskowski SW, Jayaram H, Maeder ML (2018). UDiTaS, a genome editing detection method for indels and genome rearrangements. BMC Genomics.

[28] Mardiana S, Lai J, Beavis PA, Darcy PK, House IG (2019). Switching on the green light for chimeric antigen receptor T-cell therapy. Clin Transl Immunology.

[29] Xiao L, Cen D, Gan H, Sun Y, Huang N, Xiong H, Jin Q, Su L, Liu X, Wang K (2019). Adoptive transfer of NKG2D CAR mRNA-engineered natural killer cells in colorectal Cancer patients. Mol Ther.

[30] Hu W, Wang G, Huang D, Sui M, Xu Y (2019). Cancer immunotherapy based on natural killer cells: current Progress and new opportunities. Front Immunol.

[31] Zhang C, Oberoi P, Oelsner S, Waldmann A, Lindner A, Tonn T, Wels WS (2017). Chimeric antigen receptor-engineered NK-92 cells: an off-the-shelf cellular therapeutic for targeted elimination of Cancer cells and induction of protective antitumor immunity. Front Immunol.

[32] Boroughs AC, Larson RC, Choi BD, Bouffard AA, Riley LS, Schiferle E, Kulkarni AS, Cetrulo CL, Ting D, Blazar BR, et al. Chimeric antigen receptor costimulation domains modulate human regulatory T cell function. JCI Insight. 2019;4(8):e126194. 10.1172/jci.insight.126194 PMC6538349 30869654

[33] Sommer C, Boldajipour B, Kuo TC, Bentley T, Sutton J, Chen A, Geng T, Dong H, Galetto R, Valton J (2019). Preclinical evaluation of allogeneic CAR T cells targeting BCMA for the treatment of multiple myeloma. Mol Ther.

[34] Lock D, Mockel-Tenbrinck N, Drechsel K, Barth C, Mauer D, Schaser T, Kolbe C, Al Rawashdeh W, Brauner J, Hardt O (2017). Automated manufacturing of potent CD20-directed chimeric antigen receptor T cells for clinical use. Hum Gene Ther.

[35] Qin H, Ramakrishna S, Nguyen S, Fountaine TJ, Ponduri A, Stetler-Stevenson M, Yuan CM, Haso W, Shern JF, Shah NN (2018). Preclinical development of bivalent chimeric antigen receptors targeting both CD19 and CD22. Mol Ther Oncolytics.

[36] Xu J, Chen LJ, Yang SS, Sun Y, Wu W, Liu YF, Xu J, Zhuang Y, Zhang W, Weng XQ (2019). Exploratory trial of a biepitopic CAR T-targeting B cell maturation antigen in relapsed/refractory multiple myeloma. Proc Natl Acad Sci U S A.

[37] Zah E, Lin MY, Silva-Benedict A, Jensen MC, Chen YY (2016). T cells expressing CD19/CD20 Bispecific chimeric antigen receptors prevent antigen escape by malignant B cells. Cancer Immunol Res.

[38] Ahmed N, Brawley V, Hegde M, Bielamowicz K, Kalra M, Landi D, Robertson C, Gray TL, Diouf O, Wakefield A (2017). HER2-specific chimeric antigen receptor-modified virus-specific T cells for progressive Glioblastoma: a phase 1 dose-escalation trial. JAMA Oncol.

[39] Pituch KC, Miska J, Krenciute G, Panek WK, Li G, Rodriguez-Cruz T, Wu M, Han Y, Lesniak MS, Gottschalk S (2018). Adoptive transfer of IL13Ralpha2-specific chimeric antigen receptor T cells creates a pro-inflammatory environment in Glioblastoma. Mol Ther.

[40] Posey AD, Schwab RD, Boesteanu AC, Steentoft C, Mandel U, Engels B, Stone JD, Madsen TD, Schreiber K, Haines KM (2016). Engineered CAR T cells targeting the Cancer-associated Tn-Glycoform of the membrane Mucin MUC1 control adenocarcinoma. Immunity.

[41] Majzner RG, Theruvath JL, Nellan A, Heitzeneder S, Cui Y, Mount CW, Rietberg SP, Linde MH, Xu P, Rota C (2019). CAR T cells targeting B7-H3, a pan-Cancer antigen, demonstrate potent preclinical activity against pediatric solid tumors and brain tumors. Clin Cancer Res.

[42] Helsen CW, Hammill JA, Lau VWC, Mwawasi KA, Afsahi A, Bezverbnaya K, Newhook L, Hayes DL, Aarts C, Bojovic B (2018). The chimeric TAC receptor co-opts the T cell receptor yielding robust anti-tumor activity without toxicity. Nat Commun.

[43] Lanitis E, Poussin M, Klattenhoff AW, Song D, Sandaltzopoulos R, June CH, Powell DJ (2013). Chimeric antigen receptor T cells with dissociated signaling domains exhibit focused antitumor activity with reduced potential for toxicity in vivo. Cancer Immunol Res.

[44] Feucht J, Sun J, Eyquem J, Ho YJ, Zhao Z, Leibold J, Dobrin A, Cabriolu A, Hamieh M, Sadelain M (2019). Calibration of CAR activation potential directs alternative T cell fates and therapeutic potency. Nat Med.

[45] Zhao Y, Wang QJ, Yang S, Kochenderfer JN, Zheng Z, Zhong X, Sadelain M, Eshhar Z, Rosenberg SA, Morgan RA (2009). A herceptin-based chimeric antigen receptor with modified signaling domains leads to enhanced survival of transduced T lymphocytes and antitumor activity. J Immunol.

[46] Kochenderfer JN, Yu Z, Frasheri D, Restifo NP, Rosenberg SA (2010). Adoptive transfer of syngeneic T cells transduced with a chimeric antigen receptor that recognizes murine CD19 can eradicate lymphoma and normal B cells. Blood.

[47] Gattinoni L, Speiser DE, Lichterfeld M, Bonini C (2017). T memory stem cells in health and disease. Nat Med.

[48] Hale M, Mesojednik T, Romano Ibarra GS, Sahni J, Bernard A, Sommer K, Scharenberg AM, Rawlings DJ, Wagner TA (2017). Engineering HIV-resistant, anti-HIV chimeric antigen receptor T cells. Mol Ther.

[49] Yang H, Wallace Z, Dorrell L (2018). Therapeutic targeting of HIV reservoirs: how to give T cells a new direction. Front Immunol.

[50] Kruse RL, Shum T, Tashiro H, Barzi M, Yi Z, Whitten-Bauer C, Legras X, Bissig-Choisat B, Garaigorta U, Gottschalk S (2018). HBsAg-redirected T cells exhibit antiviral activity in HBV-infected human liver chimeric mice. Cytotherapy.

[51] Ellebrecht CT, Bhoj VG, Nace A, Choi EJ, Mao X, Cho MJ, Di Zenzo G, Lanzavecchia A, Seykora JT, Cotsarelis G (2016). Reengineering chimeric antigen receptor T cells for targeted therapy of autoimmune disease. Science.

[52] Kansal Rita, Richardson Noah, Neeli Indira, Khawaja Saleem, Chamberlain Damian, Ghani Marium, Ghani Qurat-ul-ain, Balazs Louisa, Beranova-Giorgianni Sarka, Giorgianni Francesco, Kochenderfer James N., Marion Tony, Albritton Lorraine M., Radic Marko (2019). Sustained B cell depletion by CD19-targeted CAR T cells is a highly effective treatment for murine lupus. Science Translational Medicine.

